# Relationship of Age for Grade and Pubertal Stage to Early Initiation of Substance Use

**DOI:** 10.5888/pcd12.150234

**Published:** 2015-11-19

**Authors:** Rebecca N. Dudovitz, Paul J. Chung, Marc N. Elliott, Susan L. Davies, Susan Tortolero, Elizabeth Baumler, Stephen W. Banspach, Mark A. Schuster

**Affiliations:** Author Affiliations: Paul J. Chung, University of California Los Angeles, Los Angeles, California, and RAND Corp, Santa Monica, California; Marc N. Elliott, RAND Corp, Santa Monica, California; Susan L. Davies, University of Alabama, Birmingham, Alabama; Susan Tortolero, Elizabeth Baumler, University of Texas Health Science Center, Houston, Texas; Stephen W. Banspach, Division of Adolescent and School Health, Centers for Disease Control and Prevention, Atlanta, Georgia; Mark A. Schuster, Division of General Pediatrics, Boston Children's Hospital/Harvard Medical School, Boston, Massachusetts.

## Abstract

**Introduction:**

Studies suggest students who are substantially older than the average age for their grade engage in risky health behaviors, including substance use. However, most studies do not account for the distinct reasons why students are old for their grade (ie, grade retention vs delayed school entry) or for their pubertal stage. Thus, whether the association between age for grade and substance use is confounded by these factors is unknown. We sought to determine whether age, grade, or pubertal stage were associated with early substance use.

**Methods:**

Cross-sectional Healthy Passages Wave I survey data from 5,147 fifth graders and their caregivers in Alabama, California, and Texas from 2004 through 2006 were analyzed in 2014. Logistic regressions examined whether older age for grade, grade retention, delayed school entry, or pubertal stage were associated with use of any substance, cigarettes, alcohol, or other drugs.

**Results:**

Seventeen percent of fifth graders reported trying at least 1 substance. Among boys, advanced pubertal stage was associated with increased odds of cigarette, alcohol, or other drug use, whereas delayed school entry was associated with lower odds of any substance, alcohol, or other drug use. Among girls, advanced pubertal stage was associated only with higher odds of alcohol use, and delayed school entry was not associated with substance use. Neither older age for grade or grade retention was independently associated with substance use after controlling for potential confounders.

**Conclusion:**

Advanced pubertal stage may be a more important risk factor for substance use than age for grade. Pediatricians should consider initiating substance use screening earlier for patients with advanced pubertal stage.

## Introduction

In any classroom, a substantial proportion of students are significantly older than their same-grade peers, that is, they are old for grade (OFG) (1). Although some students are OFG because they repeated a grade (grade retention), others are OFG because they started school later, often because of intentionally delayed entry into elementary school (delayed school entry). Becoming OFG by grade retention or delayed school entry occurs relatively early in childhood. For instance, Byrd et al found that among OFG 17-year-old students, 77% had become OFG by third grade (2). Additionally, the number of children with delayed school entry increased during the past 40 years. A 2005 study estimated that 15% of 6-year-old children had not yet started first grade (3). This trend toward “academic redshirting” may be driven by research suggesting that students who start school older than their peers have a modest but significant educational advantage (3–5). Although decisions on school enrollment and advancement are made primarily for educational reasons (6), they may have implications for a child’s mental and behavioral health.

Numerous studies have found higher rates of high-risk behaviors, including substance use, sexual risk-taking, school infractions, and gun-carrying, among adolescent OFG students (7–11). However, in these studies, all OFG students are pooled, without differentiation by their reason for being OFG, so it is not known whether the association between being OFG and high-risk behaviors is found in grade-retained OFG students only, delayed school-entry OFG students only, or both. Given evidence that poor academic performance is associated with high-risk behaviors, grade-retained students may be more likely at baseline to adopt unhealthy behaviors (5,12). Likewise, because behavioral or academic readiness concerns might motivate delayed school entry, redshirted students may also be more likely at baseline to adopt unhealthy behaviors despite their slightly better average academic performance.

Other factors may confound the relationship between OFG and risky health behaviors. For instance, the experience of being older might place OFG students at higher risk for unhealthy behaviors than their peers because of their relatively advanced physical and social–emotional maturation, particularly with respect to the onset of puberty. Although they did not control for pubertal stage or timing, Byrd et al found that being OFG, even without grade retention, was associated with higher rates of behavior problems compared with students who were neither OFG nor grade retained (2).

In general, the association between delayed school entry and health behaviors is poorly understood. Some studies suggest that delayed school entry is a risk factor for poor academic performance, particularly among socially disadvantaged children (1) and might be associated with high-risk behaviors (2,7–9,11); however, other studies suggest that delayed school entry might lead to — or at least be a marker of factors that lead to — better academic achievement and less risky health behaviors (3–5,13). For example, parents who choose to postpone enrolling their child in school may have more financial resources (allowing an additional year of care at home or in preschool) or be more engaged in their child’s education (13). Finally, although both the reasons for being OFG and the experience of being OFG might differ between boys and girls (2), no study examines whether associations between OFG and substance use differ by sex.

Determining whether OFG is independently associated with risky health behaviors, or whether it is merely a marker of other contextual factors driving this association, is important for accurate counseling of families on school entry and advancement and for developing school policies that maximize population health. This study examined data from a preadolescent population (1) to determine the effects of age for grade and pubertal stage and to increase our understanding of the relationships among OFG, delayed school entry, grade retention, and substance use.

## Methods

In 2014, we analyzed data from Wave I (2004–2006) of Healthy Passages, a survey of 5,147 fifth graders and their primary caregivers from Los Angeles, California; Birmingham, Alabama; and Houston, Texas. At each site, we used a 2-stage probability sampling procedure in which study schools were sampled from all public schools in the geographic region with enrollment of 25 or more fifth graders. Letters were sent home with 11,532 fifth-grade students from study schools requesting permission to contact each student’s primary caregiver. Among the 6,663 students whose parents permitted the study team to contact them, both the student and one of their primary caregivers were invited to participate. Of those, 5,147 (77%) students completed the interview (14,15).

Data were collected via computer-assisted personal interviews and audio-computer–assisted self-interviews. Each child and 1 primary caregiver were interviewed separately to ensure confidentiality. The study protocol was approved by institutional review boards at each study site and at the Centers for Disease Control and Prevention.

### Measures

#### Predictors

Age for grade was calculated from each student’s birth date, as reported by the primary caregiver. To determine the association between being OFG and substance use, age for grade relative to a student’s immediate same-grade peers was measured. Thus, each student’s age was centered on the mean age for grade at his or her school after excluding 19 influential outliers (deviations of more than 2 years in schools with 35 or fewer observations). A sensitivity analysis that restored the outliers (0.4% of the data) yielded similar results.

Grade retention was assessed by student self-report. Students who indicated that they had ever repeated a grade in school were considered grade-retained.

Delayed school entry was measured indirectly by assuming that students who were older than their expected age for grade and did not have a history of grade retention entered school late. The expected age for grade was calculated from each student’s birth date and the applicable cutoff date for school entry (September 1 for Birmingham and Houston; December 1 for Los Angeles). Students who did not report repeating a grade in school but were in a lower grade than would be expected if they had started school on time and had progressed normally were considered to have delayed school entry.

Initiation of substance use has been associated with the onset of puberty (16), and pubertal stage is likely to be more advanced in OFG students. Hence pubertal stage has the potential to confound a possible association between age for grade and substance use, particularly among early adolescents. Pubertal status was measured using 2 questions that included Tanner staging pictures (pubic hair and genital development for boys; pubic hair and breast development for girls). Students selected the pictures that most closely depicted their stage of physical development, and their 2 responses were averaged to measure overall pubertal status (stage 1–5: 1 = pre-pubescent, 5 = fully mature). Correlation between the items was 0.30 for boys and 0.41 for girls, as expected given the somewhat weak correlation between the timings of androgenic hair development and sexual maturation (17). Pubertal stage varies significantly at this age (18), as indicated by a correlation of 0.24 between chronological age and Tanner self-staging.

#### Outcomes

One dichotomous (yes/no) item assessed lifetime cigarette smoking by asking students whether they had ever tried smoking cigarettes. One dichotomous item assessed past-year alcohol use by asking students whether they had taken more than a few sips of alcohol in the past 12 months, excluding drinking a few sips of alcohol for religious purposes. A dichotomous measure of whether the child had ever used another drug was derived from student responses to whether they had ever used marijuana, ever sniffed glue or used inhalants to get high, or ever used any pill or other drug to get high. Students who answered yes to any of these questions were considered to have a history of other drug use. Finally, answering yes to any of the above substance use items (cigarette, alcohol, or other drug use) was used as a dichotomous measure of overall substance use initiation.

#### Covariates

Covariates with potential associations with both age for grade and health behaviors were included.

#### School functioning

School functioning is likely associated with grade retention, delayed school entry, and substance use, making it an important potential confounder. School functioning was measured via child responses to the PedsQL School Functioning Subscale (19). This 5-item self-report subscale asks the frequency of having problems with paying attention in school, doing school work, forgetting things, missing school because of not feeling well, and missing school because of doctor visits or being in the hospital, with higher scores corresponding to better school functioning. Scores on each item range from 0 to 4 with higher scores reflecting better school function. Scores were summed and multiplied by 5 for an overall range of 0 to 100. In this sample, the subscale had an internal consistency of 0.65 and a mean of 74.1. Additionally, higher school functioning scores were associated with lower odds of having been grade-retained (odds ratio [OR] = 0.747 per 10 points, *P* < .001), supporting the construct validity of this measure.

The remaining covariates were taken from the parent survey.

#### School mobility

School mobility was measured by the number of elementary schools attended by the child. The potential effect of changing schools on risk (eg, delinquency, other problem behavior) (20) and protective (eg, school achievement, coping) (21) behaviors in children has been the focus of several studies, although results are inconsistent (22).

#### Parental involvement

Parental involvement was measured by combining 10 items from the FACES III scale with measures of parental involvement in students’ social and scholastic lives (2, 23). Items were how often the parent attended school events, volunteered at school, ate with the child, did fun things with the child, knew what the child did after school, knew what the child did during free time, knew the child’s friends, knew who the child’s best friend was, and knew the parents of the child’s friends. In this sample, the internal consistency of this measure was 0.80.

#### Sociodemographics

Data on highest household level of education and household income were used to measure socioeconomic status. Categories of missing values for annual household income and parental level of education were included because these data tend not to be missing at random (24). Additional covariates were family composition (2-parent family, single-parent family, or other), race/ethnicity, study site, interview date, whether the child had health insurance, and whether the child was born in the United States.

### Data analysis

Multivariate logistic regressions assessed whether age for grade, grade retention, delayed school entry, or pubertal stage were significantly associated with each substance use outcome.. To isolate the association between chronological age and early initiation of substance use after controlling for potential confounders, we included the linear measure of relative age for grade, an indicator for grade retention, an indicator for delayed school entry, and a linear measure of pubertal stage in the models. Collinearity among the major predictors was low, with variance inflation factor values less than 2 for all predictors of interest. In addition, these multivariate models controlled for school functioning, school mobility, parental involvement, and other sociodemographic covariates.. All analyses were performed separately for boys and girls. Finally, to determine whether delayed school-entry OFG students differed from other students with respect to contextual factors associated with substance use, a multivariate logistic regression of delayed school entry on the covariates in our model was performed. We used Stata software, version 13 (StataCorp) to account for design and nonresponse weights, clustering of children within schools, and site stratification (12).

## Results

The mean age of the sample was 10.6 years, ranging from 9.6 to 13.8 years (Table 1). For students who reported grade retention, the mean age was 11.5 years, whereas the mean age for students with delayed school entry was 11.3 years. Overall, 18% of the sample was OFG — 13% reported grade retention, and 5% delayed school entry.

**Table 1:** Demographic Characteristics of Old-for-Grade Fifth-Grade Participants (N = 5,147) in Healthy Passages Survey, Los Angeles, California; Birmingham, Alabama; and Houston, Texas, 2014

Characteristic	Total Sample, n = 5,129	Grade Retained^b^, n = 665		Delayed School Entry, n = 249	
	Value^a^	Value^a^	*P* Value^c^	Value^a^	*P* Value^c^
**Age, mean, y**	10.6	11.5	<.001	11.3	<.001
**Male**	51.1	58.9	<.001	55.6	.19
**Parental education level**					
Less than high school	23.1	36.9	<.001	25.2	.54
High school	21.4	29.9	<.001	22.5	.70
Some college	24.8	19.0	.004	15.6	.003
College graduate	28.9	11.0	<.001	36.1	.05
Missing data	1.9	3.3	.04	0.7	.32
**Household income**					
<$25,000	38.1	54.3	<.001	41.0	.46
$25,000–$49,000	24.6	24.3	.83	16.5	.006
$50,000–$99,000	16.6	8.1	<.001	14.9	.51
≥$100,000	12.4	4.8	<.001	19.0	.005
Missing data	8.6	8.6	.99	10.1	.33
**Race/ethnicity**					
White	22.1	11.6	<.001	30.8	.006
Black	29.0	31.8	.21	22.9	.06
Latino	44.5	54.3	<.001	39.9	.26
Other	4.5	2.3	<.001	6.5	.10
**Family composition**					
2-Parent family	58.1	50.2	<.001	58.0	.98
Single-parent family	37.6	42.6	.01	38.1	.89
Other	4.3	7.2	.003	3.9	.77
**No health insurance**	13.5	23.4	<.001	18.2	.09
**Born outside United States**	10.1	14.2	.005	22.4	<.001
**No. of elementary schools attended**	2.0	2.5	<.001	2.3	.001
**Parental involvement^d^**	2.3	2.2	<.001	2.3	.91
**School functioning^e^**	74.1	68.3	<.001	72.7	.28
**Pubertal stage^f^**	2.4	2.7	<.001	2.6	.006
**Substance use**					
Any	17.4	22.1	.005	15.8	.51
Cigarettes	6.6	10.3	<.001	7.3	.73
Alcohol	5.5	8.3	.01	5.0	.75
Other drug	9.8	11.8	.11	8.9	.61

a Values are percentages unless otherwise indicated.

b Grade-retained refers to students held back to repeat a grade.

c *P* values were calculated using survey-weighted linear and logistic regressions to compare means and proportions of grade-retained participants with all others and delayed school entry participants with all others. All values reflect survey weights.

d Parental involvement is the level of involvement parents have in their children’s social and scholastic lives, as measured by parents’ responses on the FACES III scale (2,23).

e School functioning is defined by the PedsQL School Function subscale (19).

f Pubertal stage is defined by Tanner self-staging (17).

Of the sample, 51.1% were male, although boys constituted 58.9% of grade-retained students and 55.6% of delayed school-entry students. Students from households earning less than $25,000 annually made up 38.1% of the sample, and 44.5% of parents did not attend college. Overall, 17.4% of students reported trying at least one substance: 6.6% reported smoking cigarettes, 5.5% drinking alcohol, and 9.8% using any other drug.

Results from the multivariate logistic regressions including age for grade, grade retention, delayed school entry, pubertal stage, and covariates are shown by sex (Table 2). Among boys, more advanced pubertal stage was associated with higher odds of using any substance (OR = 1.24; 95% confidence interval [CI], 1.05–1.46; *P* = .01), using cigarettes (OR = 1.55; 95% CI, 1.23–1.95; *P* < .001), and using alcohol (OR = 1.32; 95% CI, 1.08–1.62; *P* = .007). Neither age for grade or grade retention was significantly associated with substance use. However, delayed school entry was associated with lower odds of initiating use of any substance (OR = 0.31; 95% CI, 0.16–0.60; *P* = .001), use of alcohol (OR = 0.18; 95% CI, 0.05–0.63; *P* = .008), and use of other drugs (OR = 0.40; 95% CI, 0.18–0.90, *P* = .03).. Among girls, more advanced pubertal stage was associated only with higher odds of alcohol use (OR = 1.25, 95% CI, 1.02–1.55, *P* = .04), and there was no significant association between age for grade, grade retention, or delayed school entry and substance use initiation. Sensitivity analyses including various combinations of these predictors yielded similar results, suggesting these findings did not result from model over-specification.

**Table 2:** Association of Age for Grade With Substance Use, Fifth-Grade Participants (N = 5,147) in Healthy Passages Survey, Los Angeles, California; Birmingham, Alabama; and Houston, Texas, 2014^a^

Variable^b^	Any Substance	Cigarettes	Alcohol	Other Drug
	AOR (95% CI)	AOR (95% CI)	AOR (95% CI)	AOR (95% CI)
**Boys**				
Age for grade	1.21 (0.89–1.65)	1.38 (0.81–2.36)	1.25 (0.81–1.93)	0.94 (0.63–1.41)
Grade retention	1.03 (0.75–1.41)	0.81 (0.47–1.38)	1.33 (0.76–2.32)	1.03 (0.68–1.57)
Delayed school entry	0.31 (0.16–0.60)	0.46 (0.16–1.31)	0.18 (0.05–0.63)	0.40 (0.18–0.90)
Pubertal stage	1.24 (1.05–1.46)	1.55 (1.23–1.95)	1.32 (1.08–1.62)	1.12 (0.92–1.37)
**Girls**				
Age for grade	0.97 (0.68–1.38)	1.27 (0.75–2.15)	0.86 (0.54–1.37)	0.99 (0.64–1.52)
Grade retention	1.01 (0.68–1.51)	0.91 (0.50–1.68)	1.31 (0.72–2.36)	1.00 (0.61–1.66)
Delayed school entry	1.40 (0.70–2.81)	1.54 (0.58–4.11)	1.69 (0.63–4.54)	1.23 (0.54–2.78)
Pubertal stage	1.09 (0.94–1.26)	1.18 (0.96–1.45)	1.25 (1.02–1.55)	1.00 (0.82–1.23)

Abbreviations: AOR, adjusted odds ratio; CI, confidence interval.

a Models control for family income, highest level of parental education, family structure, race/ethnicity, whether the child had health insurance, whether the child was born in the United States, parental involvement, school mobility, school functioning, study site, and interview date.

b Age for grade is a linear measure for chronological age; grade retention is an indicator for having been held back in school; delayed school entry is an indicator for having an older age than would be expected for a fifth grader without having ever been grade retained; pubertal stage is the respondent’s self-reported Tanner stage (17) for pubertal development.

Attending more than one elementary schools (OR = 1.17 per school; 95% CI, 1.05–1.31, *P* = .004) and being born outside of the United States (OR = 2.83; 95% CI, 1.70–4.72, *P* < .001) were associated with higher odds of delayed school entry (Table 3). For race/ethnicity, being black (OR = 0.51; 95% CI, 0.34–0.80, *P* = .003) or Latino (OR = 0.51; 95% CI, 0.29–0.89, *P* = .02) was associated with lower odds of delayed school entry than being white. The odds of delayed school entry were similar for the highest-income and lowest-income families, whereas middle-income families, (ie, those earning between $25,000 and $99,000 per year) had the lowest odds of delayed school entry (OR = 0.63; 95% CI, 0.42–0.93, *P* = .02), compared with families earning $100,000 or more per year. Sex did not significantly predict delayed school entry.

**Table 3:** Predictors of Delayed School Entry, Fifth-Grade Participants, Healthy Passages Survey, Los Angeles, California; Birmingham, Alabama; and Houston, Texas, 2014^a^

Predictors	Odds Ratio (95% CI)
**Parental involvement^b^**	0.90 (0.55–1.48)
**No. of elementary schools attended**	1.17 (1.05–1.31)
**Family composition**	
2-Parent household	1 [Reference]
Single-parent household	1.22 (0.85–1.76)
Other family composition	0.96 (0.46–2.04)
**Household income**	
<$25,000	1 [Reference]
$25,000–$49,999	0.64 (0.41–1.01)
$50,000–$99,999	0.77 (0.43–1.37)
≥$100,000	1.14 (0.65–1.99)
**Parental level of education**	
Less than high school	1 [Reference]
High school	1.17 (0.72–1.90)
Some college	0.63 (0.36–1.11)
College graduate	1.04 (0.54–2.01)
**No health insurance**	1.30 (0.84–2.01)
**Born outside United States**	2.83 (1.70–4.72)
**Male **	1.17(0.88–1.56)
**Race/ethnicity**	
White	1 [Reference]
Black	0.51 (0.34–0.80)
Latino	0.51 (0.29–0.89)
Other	0.99 (0.54–1.80)

Abbreviation: CI, confidence interval.

a Model also controls for site, interview date, and missing values for income (8.6%) and parent education (1.9%).

b Parental involvement is the level of involvement parents have in their children’s social and scholastic lives, as measured by parents’ responses on the FACES III scale (2,23).

## Discussion

These findings indicate that the association between being OFG and substance use differs depending on the reason for being OFG. Delayed school entry did not confer a higher risk for substance use initiation, particularly for boys. Given that approximately 7% to 15% of US children have delayed school entry each year (3,25), these findings are reassuring for a substantial percentage of US children. However, students with delayed school entry are a heterogeneous group; further studies are needed to determine whether associations with substance use vary by reason for delayed school entry.

Pubertal stage emerged as a significant predictor of initiation of any substance use, cigarette use, and alcohol use for boys and of alcohol use initiation for girls. These results are consistent with other findings linking the timing and pace of puberty with early and midadolescent drug use (16,26) and the social and physiologic changes that accompany adolescence (27). Socially, advanced pubertal stage and early pubertal timing are associated with having more deviant peers (28). Physiologically, increased testosterone levels enhance sensation-seeking and reduce impulse control such that boys, in particular, may engage in more risky behaviors (27,29). This may explain the significant association between advanced pubertal stage and more types of substance use among boys than girls in this study. After accounting for pubertal stage, grade retention (often assumed to identify a high-risk population) was not independently associated with substance use.

These findings have important implications for substance use screening. Clinicians should consider a preadolescent’s pubertal stage, rather than merely their chronological age or school grade, when deciding at what point to initiate screening for risky health behaviors. Current recommendations to initiate substance use screening based on a child’s chronological age may result in late detection of risky behaviors for children with early or more advanced pubertal timing (3,30). For example, in this sample, 12% of fifth graders who self-identified as having a Tanner I pubertal stage reported trying at least one substance, but more than 26% of those who self-identified as having a Tanner V pubertal stage tried at least one substance. Given the importance of active screening for risky health behaviors, coupled with the negative impact of early substance use initiation, attention to pubertal stage may help clinicians avoid a missed opportunity to identify and counsel young patients at increased risk for substance use.

This study is limited by its cross-sectional design and thus did not determine whether the associations with substance use initiation vary over time or whether advanced pubertal stage causes early initiation of substance use. Although reverse causality is highly unlikely, we cannot exclude the possibility that unmeasured confounding variables are associated with both advanced pubertal stage and early initiation of substance use. Second, these findings may not generalize to a national sample, because Healthy Passages included only preadolescents from 3 large US cities and their surrounding communities. However, the cities span different regions of the country, and the sample is demographically diverse. Third, because this study focused on fifth graders, there were low rates of substance use initiation, limiting the power to detect small differences in these behaviors across groups. Thus, some of the nonsignificant findings would possibly be significant in a larger or older sample. Additionally, the drivers of substance use initiation are likely to change as students mature. However, studying substance use in this population provides insight into the age of initiation of these health behaviors and when the effects of age for grade and pubertal stage might emerge.

Despite these limitations, the findings have important implications for children’s health. Parents and education policy makers may be reassured that delayed school entry may not be a risk factor for early initiation of substance use. Additionally, the finding that pubertal stage, independent of chronological age, may be an important driver of substance use may help clinicians decide when to begin screening for risky health behaviors among preadolescent patients.
